# Therapeutic Potential of Oridonin and Its Analogs: From Anticancer and Antiinflammation to Neuroprotection

**DOI:** 10.3390/molecules23020474

**Published:** 2018-02-22

**Authors:** Jimin Xu, Eric A. Wold, Ye Ding, Qiang Shen, Jia Zhou

**Affiliations:** 1 Chemical Biology Program, Department of Pharmacology and Toxicology, University of Texas Medical Branch, Galveston, TX 77555, USA; jimxu@utmb.edu (J.X.); eawold@utmb.edu (E.A.W.); dingye515@outlook.com (Y.D.); 2 Department of Clinical Cancer Prevention, Division of Cancer Prevention and Population Sciences, The University of Texas MD Anderson Cancer Center, Houston, TX 77030, USA; qshen@mdanderson.org

**Keywords:** oridonin, anticancer, antiinflammation, neuroprotection, Alzheimer’s disease

## Abstract

Oridonin, a diterpenoid natural product commonly used in East Asian herbal medicine, is garnering increased attention in the biomedical community due to its extensive biological activities that include antitumor, anti-inflammatory, antimicrobial, hepatic fibrosis prevention, and neurological effects. Over the past decade, significant progress has been made in structure activity relationship and mechanism of action studies of oridonin for the treatment of cancer and other diseases. This review provides a brief summary on oridonin and its analogs in cancer drug discovery and antiinflammation and highlights its emerging therapeutic potential in neuroprotection applications.

## 1. Introduction

**Figure 1:**
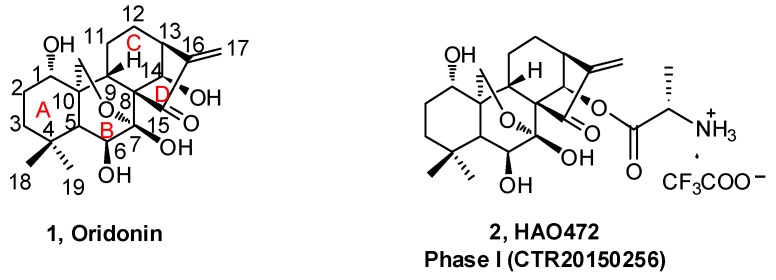
The structures of oridonin and HAO472.

Natural products derived from animals, plants, and microbes have played an important role in the treatment of human diseases since the dawn of medicine. Natural products have high chemical diversity, bio-compatible characteristics, and other molecular properties that make them advantageous as lead scaffolds for drug discovery [1,2,3,4]. A detailed analysis of first-in-class drugs approved by the US Food and Drug Administration (FDA) from 1999 to 2013 revealed 31 (28%) of those drugs (112) were natural products or their derivatives [5]. *Rabdosia rubescens*, a herbal plant also known as *Donglingcao*, was used in East Asian traditional medicine for the treatment of inflammation and cancer [6]. Oridonin (**1**, Figure 1), an *ent*-kaurane diterpenoid isolated from *Rabdosia rubenscens*, was first identified as an antitumor compound in 1967 [7,8]. It has since attracted considerable attention due to its various pharmacological and physiological properties including antitumor [9,10,11,12,13,14,15,16,17], anti-inflammatory [18,19,20], antimicrobial [21], and hepatic fibrosis prevention actions [22,23,24,25], and its effects in the central nervous system (CNS) [26,27,28]. Over the past decade, significant progress has been made in structural optimization and mechanism of action studies of oridonin for the treatment of cancer and other diseases [29,30,31]. For example, Hengrui Medicine Co. Ltd. (Lianyungang, China) was recently given approval to advance HAO472 [32] (**2**, l-alanine-(14-oridonin)ester trifluoroacetate, Figure 1) into a Phase I human clinical trial (CTR20150246; www.chinadrugtrails.org.cn) in China for the treatment of acute myelogenous leukemia. In this review, we will first provide a summary of oridonin, its analogs, and their promising potential in cancer drug discovery and antiinflammation, and second, we will highlight its emerging therapeutic potential for neuroprotection. To our knowledge, this will be the first review article in the field that discusses the oridonin class of compounds as potential neurotherapeutics.

## 2. Oridonin and Its Analogs for Cancer Drug Discovery

**Figure 2:**
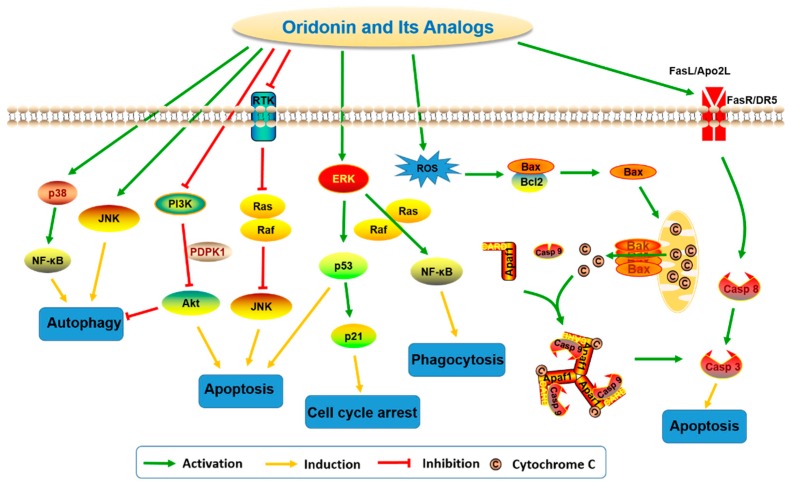
Oridonin regulates multi-signaling pathways related to autophagy, apoptosis, phagocytosis, and cell cycle arrest.

The antitumor activity of oridonin has been widely investigated and evidence suggests that oridonin may effectively inhibit the proliferation of multiple cancer cell types, including human breast cancer [9], gastric cancer [10], leukemia [11], gallbladder cancer [12], cervical carcinoma [13], and hepatocellular carcinoma [14]. Previous studies have revealed mechanisms by which oridonin can trigger autophagy, enhance phagocytosis, arrest cell cycle progression, and promote apoptosis by modulation of relevant signaling pathways (Figure 2) associated with the regulation of intracellular reactive oxygen species (ROS), Bcl-2/Bax, p53/p21, JNK, nuclear factor-kappa B (NF-κB), MAPK, PI3K, and fatty acid synthesis pathways [30,31,33]. 

In a human prostate cell line, DU-145, oridonin upregulates p53 and Bax and downregulates Bcl-2 expression in a dose-dependent manner [34]. In Hela cells, oridonin-induced autophagy is negatively regulated by Ras but positively regulated by p38 and JNK MAPKs [13]. Additionally, in HepG2 cells, oridonin was reported to increase the expression levels of p-JNK, p-p38, p-p53, and p21 and elevate the level of cyclin B1/p-Cdc2 (Tyr15) complex, which results in G2/M cell cycle arrest and apoptosis through MAPK and p53 pathways [35]. Oridonin also induces apoptosis via inhibiting PTK-mediated Ras-Raf-JNK [36] and PI3K-Akt [37] survival pathways in L929 and cervical carcinoma Hela cells, respectively. Moreover, oridonin can trigger apoptosis through activating both classic extrinsic pathways, such as Fas/FasL and Apo2L/DR5-mediated signaling pathways, and mitochondrial-mediated intrinsic pathways in several cancer cells [38,39,40,41]. In U937 cells, oridonin was reported to activate NF-κB via Ras/Raf1/ERK signaling pathway-dependent IκBα degradation and subsequently regulate oridonin-enhanced phagocytosis [42]. In colorectal cancer cells, oridonin has been shown to increase the intracellular hydrogen peroxide level and reduce the glutathione content in a dose-dependent manner [43]. It was also reported that oridonin induces a rapid and significant generation of ROS in L929 cells and subsequently upregulates the expression of phospho-p53 and increases expression ratio of Bax/Bcl-2 [44]. Modulations of these pathways in different cell models may explain the broad range of anticancer activities of oridonin.

Although oridonin has a unique, relatively safe, and extensive anticancer profile, its clinical development for cancer therapy has historically been hindered by its moderate potency, limited aqueous solubility, and poor bioavailability. To overcome these obstacles and yield oridonin analogs with increased druglikeness, iterative medicinal chemistry efforts have been made by multiple research groups and a portion of that work is discussed herein. Over the past several years, our group has synthesized a series of oridonin derivatives primarily focused on A-ring system modifications. 

**Figure 3:**
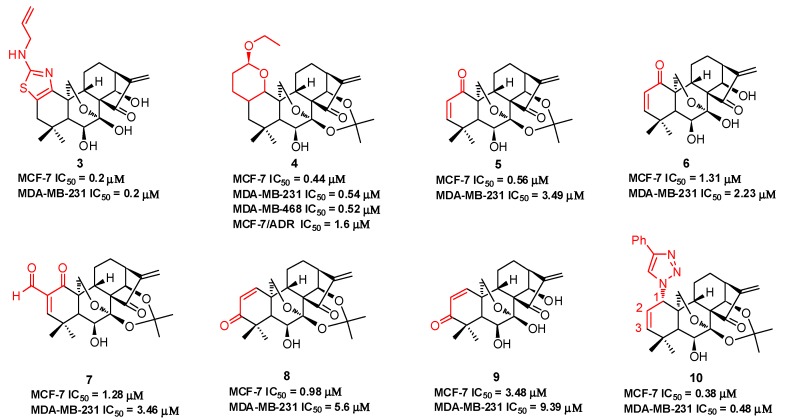
Diversified modifications on A-ring of oridonin.

Compound **3** (Figure 3), with an *N*-allyl substituted thiazole moiety, exhibits potent antiproliferative activities against human breast cancer MCF-7 (IC_50_ = 0.2 μM) and MDA-MB-231 (IC_50_ = 0.2 μM) cells, which are approximately 33-fold and 147-fold more potent than oridonin, respectively [45]. The aqueous solubility of **3** has been significantly improved with a saturated concentration of 42.4 mg/mL, which is approximately 32-fold better than that of oridonin (1.29 mg/mL). Moreover, **3** significantly suppresses MDA-MB-231 xenograft tumor growth in vivo (5 mg/kg, ip, tumor growth inhibition >66%), while oridonin shows no significant efficacy at the same dose. Among the dihydropyran-fused derivatives, **4** shows the highest inhibition potency against MCF-7 (IC_50_ = 0.44 μM), MDA-MB-231 (IC_50_ = 0.54 μM), and MDA-MB-468 (IC_50_ = 0.52 μM) cell lines and an improved ability to overcome chemoresistance in a MCF-7/ADR cell line (IC_50_ = 1.6 μM) [46]. Our group also synthesized a series of dienone derivatives of oridonin with additional α,β-unsaturated ketone system diversely installed in the A-ring [47]. Dienone analogues **5**–**9** display significant antiproliferative effects relative to oridonin against MCF-7 and MDA-MB-231 cells with low micromolar to submicromolar potency. Compared to oridonin, **8** shows lower toxicity in normal mammary epithelial cells and increased antitumor efficacy at a dose of 5.0 mg/kg (ip, growth inhibition >55%) with no significant loss of body weight in an MDA-MB-231 xenograft tumor model. Our group developed efficient and concise synthetic approaches to rapidly and diversely introduce azide functionalities at the C-1, C-2, or C-3 positions of oridonin in a highly regio- and stereospecific manner. Subsequent functionalization of these azides through click chemistry yielded triazole derivatives. These derivatives with 1,2,3-triazole installed in the A-ring system exhibit significantly improved activities against breast cancer cells compared to oridonin. Among them, 1-triazole derivative **10** displays the most potent inhibitory activities against MCF-7 (IC_50_ = 0.38 μM) and MDA-MB-231 (IC_50_ = 0.48 μM) cell lines [48].

**Figure 4:**
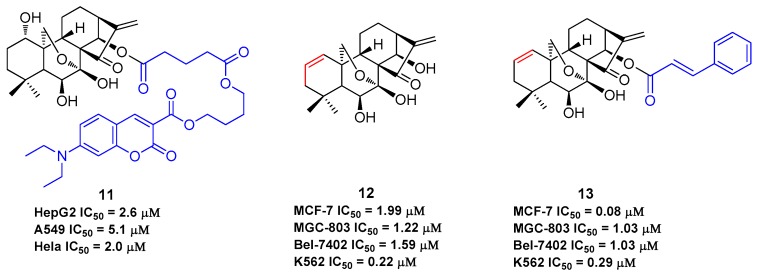
The structures of C-1 and C-14 modified oridonin derivatives.

The B-ring is inert due to the low reactivity of the 7-hydroxy group and the hydrogen bond of 6-hydroxy group with the 15-carbonyl group. The α,β-unsaturated ketone in the D-ring is the active pharmacophore of oridonin, and studies have shown that reduction or opening will significantly reduce the antiproliferative effect of oridonin [49,50,51]. Alternatively, the esterification of the hydroxyl in the C-ring is an efficient way to enhance the antiproliferative activity of oridonin other than the modifications on A-ring. HAO472 (Figure 2) was designed with an alanine ester trifluoroacetate at the C-14 position to improve its aqueous solubility (i.e., 165 mg/mL). HAO472 was said to maintain the anticancer activities of oridonin (data not disclosed), while also being less likely to cause vascular injury [32,52]. Thus, in China, HAO472 has been advanced into Phase I human clinical trials for the treatment of acute myelogenous leukemia (80–320 mg/d, iv, CTR20150246). Xu and colleagues designed and synthesized a fluorescent oridonin probe **11** (Figure 4) using a linker to connect the 14-hydroxyl group of oridonin with a coumarin moiety [49]. When tested, **11** exhibited more potent antiproliferative activities compared to oridonin in HepG2 (IC_50_ = 2.6 μM), A549 (IC_50_ = 5.1 μM) and Hela (IC_50_ = 2.0 μM) cell lines. **11** was used to confirm that a mitochondrial pathway is involved in oridonin-mediated apoptosis and that cytochrome C plays an important role in the oridonin-mediated apoptotic process. Compound **13**, bearing 1-ene and a *trans*-cinnamic acid moiety on the 14-position designed and synthesized from compound **12**, is 200-fold (IC_50_ = 0.08 μM) more potent than oridonin against MCF-7 cancer cells [53]. **13** significantly decreased tumor volume and reduced tumor weight by 69.8% at a dose of 20 mg/kg/day (iv) in an MCF-7 breast cancer xenograft nude mice model, which was greater than that of the positive control, cyclophosphamide (64.6%).

**Figure 5:**
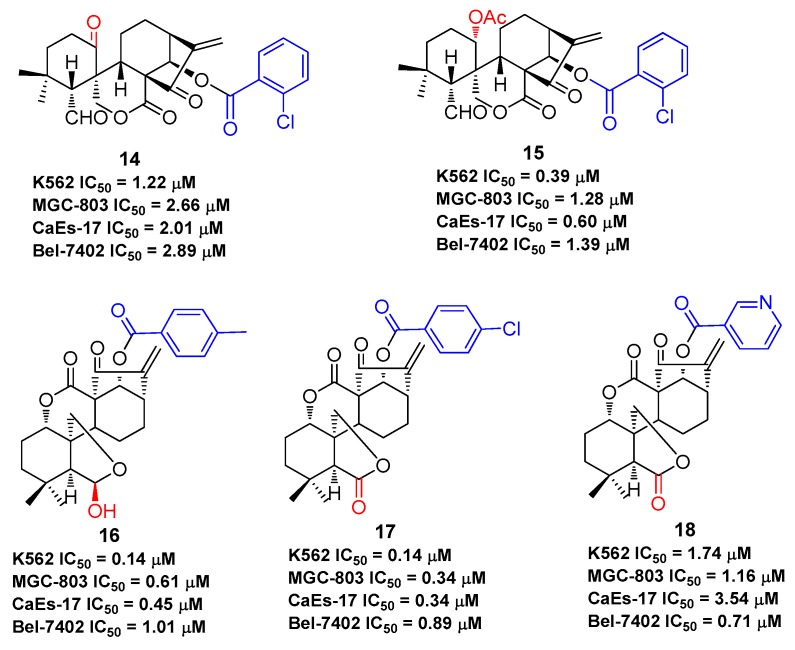
Spirolactone-type and enmein-type diterpenoid derivatives generated from oridonin.

As oridonin is abundant in natural sources and is commercially available, it can also be used as an advantageous starting material to semi-synthesize other types of diterpenoid derivatives that are otherwise rare [54,55,56,57,58,59], such as spirolactone-type diterpenoid and enmein-type diterpenoid derivatives. Spirolactone-type diterpenoid derivatives **14**–**15** [54,55] and enmein-type diterpenoid derivatives **16**–**18** [56,57], synthesized from oridonin by oxidative rearrangements around C-6 and C-7 positions, showed improved antiproliferative activities against a panel of human cancer cell lines (Figure 5). Administration of salts of water-soluble compound **18** at a dose of 40 mg/kg was found to exhibit greater anti-gastric cancer effects (ip, growth inhibition = 64.8%) when compared to oridonin (ip, growth inhibition = 37.3%) in mice [57]. The synthesis of these *ent*-kaurane diterpenoid derivatives with simpler structures and retained bioactivities serves as another key research direction for oridonin modification and diversification. The antiproliferative activities of compounds **1**–**18** against various human cancer cell lines are summarized in Table 1, which indicates that oridonin is a privileged scaffold with chemical space for diverse structural optimization and drug property enhancement. 

## 3. Antiinflammation Effects of Oridonin and Its Analogs

**Figure 6:**
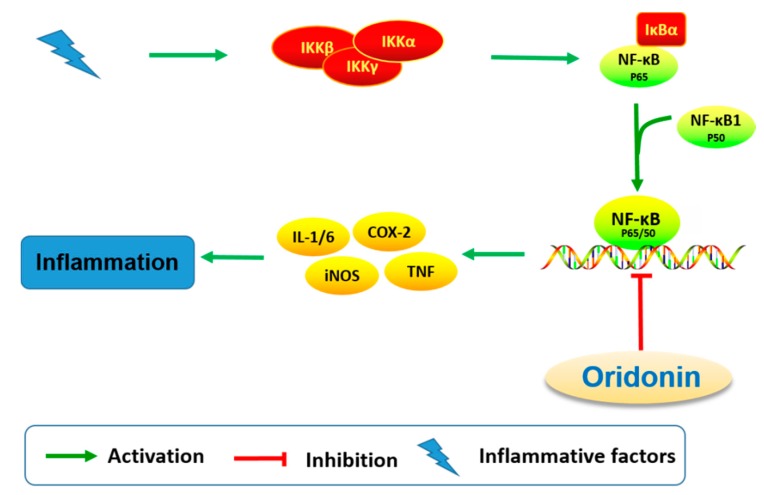
Oridonin regulates signaling pathway related to inflammation.

The effect on immune and pro-inflammatory mediators is another important bioactivity of oridonin (Figure 6). Studies have shown that oridonin can promote the differentiation of T cells towards CD4+/CD5+ Tregs, increase the secretion of IL-10, and modulate the Th1/Th2 balance via inducing HO-1 [19]. The effect of oridonin on intracellular tumor necrosis factor-α (TNF-α) expression was investigated and results showed that oridonin enhances endogenous pro-TNF-α expression and increases its upstream protein IκB phosphorylation [60]. Oridonin was reported to suppress the expression of inducible nitric oxide (iNOS) and cyclooxygenase-2 (COX-2) by inhibiting NF-κB DNA binding activity in HepG2, RAW264.7, and Jurkat cells [61,62]. Several groups have shown that oridonin and its water-soluble derivative (HAO472, Figure 1) might ameliorate TNBS-induced colitis by decreasing Th1/Th17 via inhibiting NF-κB signaling, subsequently reducing TNF-α, TNF-γ, IL-17A, iNOS/COX-2, and lymphocyte proliferation [52,63].

Additionally, it has been reported that oridonin and derivatives **7** (Figure 3) and **12** (Figure 4) exhibited anti-fibrogenic activities for the treatment of hepatic fibrosis [22,23,24,25]. The anti-fibrogenic effects of oridonin, **7** and **12** were investigated in the activated human LX-2 and rat HST-T6 stellate cell lines. The results showed that **7** and **12** significantly inhibited LX-2 cell proliferation in a dose- and time-dependent manner with IC_50_ values of 0.7 μM and 0.49 μM for 48 h, which were respectively 10-fold and 15-fold higher potency than oridonin (7.5 μM). Similar results were observed for **7** and **12** when compared to oridonin in HSC-T6 cells. However, no significant antiproliferative effects were observed on the human hepatocyte cell line C3A. These two derivatives were found to induce LX-2 cell apoptosis and S-phase cell cycle arrest and were associated with the activation of p53, p21, and cleaved caspase-3. It was also shown that **7** and **12** may mitigate endogenous production of α-SMA and ECM proteins type I collagen and fibronectin and inhibit TGF-β induced type I collagen and fibronectin production at much lower concentrations compared to oridonin. Thus, oridonin and its derivatives may hold great potential as antifibrogenic agents for the treatment of hepatic fibrosis.

## 4. Neuroinflammation and Neuroprotection Activities

**Figure 7:**
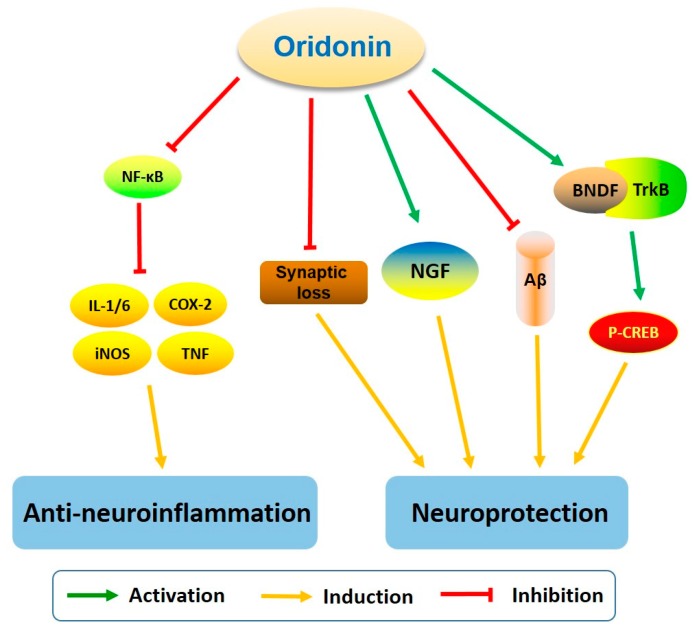
Oridonin regulates signaling pathways related to anti-neuroinflammation and neuroprotection.

Based on the anti-inflammatory properties of oridonin, its effects on neuroinflammation have been investigated by several research groups [27,64]. Microglia is regarded as the resident macrophage-like cell in the CNS, and can be activated by brain injury, infection, and various neuroinflammatory stimuli, consequently releasing proinflammatory and cytotoxic factors including nitric oxide (NO), TNF-α, interleukin-1β (IL-1β), interleukin-6 (IL-6), ROS, and eicosanoids [65,66,67,68,69]. Microglial activation has been observed in many neurological disorders and is noteworthy for its inflammatory and/or neurotrophic effects [69,70,71]. In LPS-activated microglia, oridonin pretreatment inhibits the release of proinflammatory mediators including NO, TNF-α, IL-1β, and IL-6 [64]. Suppression of proinflammatory mediators is accompanied by the inhibition of NF-κB DNA binding activity. Additionally, oridonin upregulates the expression of nerve nuclear growth factor (NGF), an essential neurotrophic factor for neuron survival and differentiation. These findings suggest that oridonin may have anti-neuroinflammatory and neuroregulatory effects (Figure 7) through modulation of multiple microglial pathways. 

Oridonin was reported to suppress microglia and astrocyte activation in the hippocampus of the Aβ_1–42_ induced Alzheimer’s disease (AD) mouse model [27]. A range of activities were reported including decreasing the mRNA levels of IL-1β, IL-6, COX-2, iNOS, TNF-α, and MCP-1, upregulating the expression of IL-10, inhibiting NF-κB p65 nuclear translocation via attenuating Aβ_1–42_ induced IκBα phosphorylation and degradation, attenuating mitochondrial dysfunction, and reducing cognitive impairment in an Aβ_1–42_ induced AD mouse model [27]. In conclusion, this study has provided evidence that oridonin and its new analogues may inhibit neuroinflammation and attenuate memory deficits induced by Aβ_1–42_.

β-Amyloid (Aβ)-mediated synaptic dysfunction plays a critical role in the pathophysiology of AD, but the underlying mechanisms for this process remain unknown [72,73,74,75]. Xu and colleagues found that oridonin diminished synaptic dysfunction induced by Aβ_1–42_ in vivo and in vitro and rescued the dendritic morphological changes observed in the hippocampus of an AD mouse model. In addition, oridonin increased the expression of PSD-95 and synaptophysin and ameliorated the Aβ-induced reduction of mitochondrial activity in the synaptosomes of an AD mouse model [26]. The expression of BDNF and its receptor TrkB is ubiquitous in the brain, and the BDNF/TrkB signaling pathway has been shown to mediate the survival and differentiation of neurons, long-term potentiation, as well as plasticity [76,77]. In addition, the BDNF/TrkB pathway has been shown to play a modulatory role in learning and memory [78]. Oridonin was found to activate the BDNF/TrkB pathway and increase p-CREB expression in the hippocampus of the Aβ-induced AD mouse model, providing insight to a possible mechanism for its neuroprotective effects [26]. Additionally, in the Morris water maze test, oridonin suppressed escaping latency and searching distance and increased the number of platform crosses in the AD mouse model. These results support that oridonin can attenuate synaptic loss and promote behavioral measures in an Aβ_1–42_ induced AD mouse model.

## 5. Oridonin for Neurodegenerative Diseases

Neurodegenerative disorders are a heterogeneous group of diseases that display diverse etiologies and may impact both the CNS and the peripheral nervous system (PNS) [79,80,81]. Major neurodegenerative diseases include AD, Parkinson’s disease (PD), Huntington’s disease, multiple sclerosis, and the prion diseases. Characteristic symptoms of these diseases may include anxiety, depression, motor dysfunction, memory loss, and cognitive impairment. The causes of neurodegenerative diseases are highly diverse and may include both hereditary or environmental factors and toxic, metabolic, or infectious processes [82]. Neuronal cell damage or death is an important factor in the progression of various neurodegenerative disorders. Thus, oxidative stress, neuroinflammation, mitochondrial dysfunction, and apoptosis are major pathways responsible for neurodegeneration [83]. Several transcription factors play a role in the pathophysiology of neuronal cell damage including Nrf2, NF-κB, MAPKs, CREB, Wnt, JAK/STAT, and TLR-4, etc. [84,85,86]. Multiple therapeutic options are available that attempt to slow disease progression or control disease symptoms, such as dopaminergic treatments, acetylcholinesterase inhibitors, NMDA receptor antagonists, antipsychotic drugs, and brain stimulation [87,88,89,90]. In addition, riluzole, non-steroidal anti-inflammatory drugs, CERE-120, and caffeine A2A receptor antagonists have been used to reduce the risk of neurodegenerative diseases onset [91]. However, none of these therapies has been effective in halting the progression of neurodegenerative diseases such as AD and PD, due to their complex pathological underpinnings. The long-term use of these drugs may also produce various negative side effects. Hence, there is a need to develop safer, multi-targeted, and more effective drugs for the treatment of neurodegenerative diseases [92,93].

**Figure 8:**
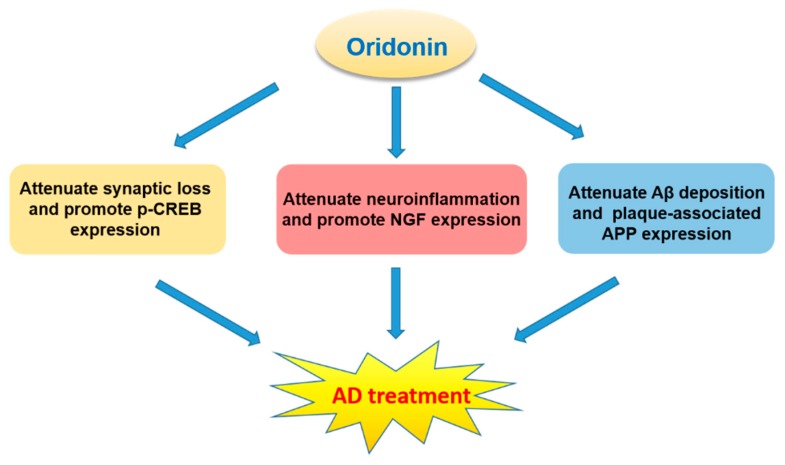
The characteristics of oridonin and its potential for AD treatment.

Oridonin was found to inhibit LPS-activated microglia inflammation and Aβ_1–42_ induced neuroinflammation, prevent synaptic loss, suppress the NF-κB pathway, and activate BDNF/TrkB/CREB and Nrf2 signaling pathways [26,27,64,94,95,96,97]. These neuroprotective effects suggest that oridonin may hold promise for the treatment of neurodegenerative diseases, especially AD (Figure 8). 

AD, the primary cause of dementia, is an irreversible neurodegenerative disorder with progressive cognitive dysfunction, memory impairment, and behavioral maladaptions. The pathological features of AD are comprised of Aβ plaques (deposition of extracellular Aβ) and neurofibrillary tangles (NFTs, accumulation of intracellular hyperphosphorylated tau protein). The interesting possibility for oridonin to treat AD has already been investigated in animal models by several groups. For instance, oridonin attenuates memory and cognitive deficits in Aβ_1–42_ induced AD mouse models [26,27]. An oral administration of an oridonin suspension significantly attenuated Aβ aggregation, plaque-associated APP expression, and microglial activation in both the cortex and hippocampus of transgenic APP/PS1 mice at 5 months of age [28]. Further, injection of an oridonin-nanoemulsion suppressed deficits in nesting (an important affiliative behavior) and social interaction. These pathological and behavioral effects of oridonin may be due to its polypharmacology and to its modulation of multiple mechanisms/factors including reduced inflammatory activation of glial cells and immune cells, decreased Aβ deposition and APP expression directly or indirectly, as well as possible neuroprotective effects via modulating microglial function and reducing local production of proinflammatory factors. With continued research, oridonin holds potential to be developed as a therapeutic option for human AD or other neurodegenerative disorders.

## 6. Conclusions and Future Directions

Oridonin, a natural product commonly used in East Asian herbal medicine, has drawn increased attention in recent years due to its extensive biological activities and potential in the treatment of various diseases. Its unique, relatively safe, and remarkable anticancer pharmacological profile are noteworthy for drug discovery campaigns. A number of oridonin derivatives were designed and synthesized to pursue more potent and drug-like candidates for cancer therapy [30]. However, the exact mechanisms by which oridonin exerts these activities were inadequately understood. During the process of investigating oridonin’s mechanism of action, a variety of potential targets and signaling pathways associated with oridonin have been identified [31]. Recently, oridonin was found to ameliorate TNBS-induced colitis and inhibit HSC proliferation and fibrogenesis [25,63]. Several studies have shown that oridonin inhibits neuroinflammation, prevents synaptic loss, and regulates several targets and signaling pathways involved in the pathophysiology of neurodegenerative diseases [26,27,64]. In addition, it may ameliorate neuropathological changes and behavioral deficits in a mouse model of cerebral amyloidosis [28]. These results suggest oridonin may have the potential to treat human AD or other neurodegenerative disorders. 

Oridonin displays limited aqueous solubility, low bioavailability via oral administration (*F* = 4.3%) or intraperitoneal injection (*F* = 12.6%), and high first-pass effects [45,98]. Although high lipophilicity is favorable for blood-brain barrier (BBB) permeability, limited aqueous solubility and bioavailability will decrease resultant therapeutic effects in vivo. These properties of oridonin will hamper its further clinical development as a neuroprotective agent. The accumulated SAR studies show that modifications on the A-ring system and C-14 position of oridonin may significantly improve its biological activities and aqueous solubility. Compound **3** with a thiazole fused A-ring and an additional nitrogen-containing side chain displays improved potency and aqueous solubility [45]. In addition, the introduction of hydrophilic groups (e.g., HAO472) or PEGylation at the C-14 position could also be a promising method to improve the absorption and distribution properties of oridonin derivatives [32,99,100]. Another useful strategy is the use of nanotechnology-based drug delivery approaches that may enhance drug solubility and bioavailability, improve permeability, and control drug release [101,102,103,104,105,106,107,108]. Additionally, nanostructured carriers such as nanosuspension, nanogels, and nanoparticles may prove to be an interesting strategy to afford a safe and effective delivery vehicle to overcome oral and CNS barriers [101]. In short, more attention should be directed towards the enhancement of pharmacokinetic properties in developing oridonin and its derivatives as neuroprotective agents. We believe that oridonin and its analogs have the potential to extend their application from anticancer and antiinflammation to neuroprotection, and may open new avenues to potential neurotherapeutics that can eventually benefit the patients with CNS disorders.

## Figures and Tables

**Table 1:** The antiproliferative effects of compounds **1**–**18** against various human cancer cell lines.

Compd.	IC_50_	Ref.
Oridonin (**1**)	MCF-7: 6.6 μM; MDA-MB-231: 29.4 μM; MDA-MB-468: 5.3 μM; MCF-7/ADR: 34.8 μM; HepG2: 15.2 μM; MGC-803: 9.06 μM; Bel-7402: 5.41 μM; K562: 4.33 μM	[45,46,47,48,49,53]
HAO472 (**2**)	Not disclosed Phase I (CTR20150246) in China: leukemia	*
**3**	MCF-7: 0.2 μM; MDA-MB-231: 0.2 μM; AsPC1: 1.1 μM; Panc-1: 1.1 μM; DU145: 1.2 μM	[45]
**4**	MCF-7: 0.44 μM; MDA-MB-231: 0.54 μM; MDA-MB-468: 0.52 μM; MCF-7/ADR: 1.6 μM	[46]
**5**	MCF-7: 0.56 μM; MDA-MB-231: 3.49 μM; MCF-7/ADR: 5.03 μM	[47]
**6**	MCF-7: 1.31 μM; MDA-MB-231: 2.23 μM; MCF-7/ADR: 5.82 μM	[47]
**7**	MCF-7: 1.28 μM; MDA-MB-231: 3.46 μM; MCF-7/ADR: 6.55 μM	[47]
**8**	MCF-7: 0.98 μM; MDA-MB-231: 5.60 μM; MCF-7/ADR: 6.02 μM	[47]
**9**	MCF-7: 3.48 μM; MDA-MB-231: 9.39 μM	[47]
**10**	MCF-7: 3.48 μM; MDA-MB-231: 9.39 μM	[48]
**11**	HepG2: 2.6 μM; A549: 5.1 μM; Hela: 2.0 μM	[49]
**12**	MCF-7: 1.99 μM; MGC-803: 1.22 μM; Bel-7402: 1.59 μM; K562: 0.22 μM	[53]
**13**	MCF-7: 0.08 μM; MGC-803: 1.03 μM; Bel-7402: 1.03 μM; K562: 0.29 μM	[53]
**14**	K562: 1.22 μM; MGC-803: 2.66 μM; CaEs-17: 2.01 μM; Bel-7402: 2.89 μM	[54]
**15**	K562: 0.39 μM; MGC-803: 1.28 μM; CaEs-17: 0.60 μM; Bel-7402: 1.39 μM	[55]
**16**	K562: 0.14 μM; MGC-803: 0.61 μM; CaEs-17: 0.45 μM; Bel-7402: 1.01 μM	[56]
**17**	K562: 0.14 μM; MGC-803: 0.34 μM; CaEs-17: 0.34 μM; Bel-7402: 0.89 μM	[57]
**18**	K562: 1.74 μM; MGC-803: 1.16 μM; CaEs-17: 3.54 μM; Bel-7402: 0.71 μM	[57]

* http://www.chinadrugtrials.org.cn/.

## Abbreviations

The following abbreviations are used in this manuscript:

α-SMA	α-Smooth muscle actin
APP	Amyloid precursor protein
Bax	Bcl-2-associated X protein
Bcl-2	B-cell lymphoma 2
BDNF	Brain-derived neurotrophic factor
CREB	cAMP response element-binding protein
ECM	Extracellular matrix
HSC	Hepatic stellate cell
ip	Intraperitoneal
iv	Intravenous
JAK	Janus kinase
JNK	c-Jun N-terminal kinase
MAPK	Mitogen-activated protein kinase
MCP-1	Monocyte chemotactic protein 1
NMDA	*N*-Methyl-d-aspartic acid
Nrf2	Nuclear factor erythroid-derived 2 (NFE2) related factor 2
p21	p21Cip1 protein
p53	Tumor protein p53
PI3K	Phosphatidylinositide 3-kinases
SAR	Structure-activity relationship
STAT	Signal transducer and activator of transcription
TLR-4	Toll-like receptor 4
TNBS	2,4,6-Trinitrobenzenesulfonic acid
TrkB	Tropomysin receptor kinase B
